# Characterization of reference genes for RT-qPCR in the desert moss *Syntrichia caninervis* in response to abiotic stress and desiccation/rehydration

**DOI:** 10.3389/fpls.2015.00038

**Published:** 2015-02-05

**Authors:** Xiaoshuang Li, Daoyuan Zhang, Haiyan Li, Bei Gao, Honglan Yang, Yuanming Zhang, Andrew J. Wood

**Affiliations:** ^1^Key Laboratory of Biogeography and Bioresource in Arid Land, Xinjiang Institute of Ecology and Geography – Chinese Academy of SciencesÜrümqi, China; ^2^Department of Plant Biology, Southern Illinois UniversityCarbondale, IL, USA

**Keywords:** *Syntrichia caninervis*, quantitative real-time PCR, reference gene, geNorm, NormFinder, RefFinder

## Abstract

*Syntrichia caninervis* is the dominant bryophyte of the biological soil crusts found in the Gurbantunggut desert. The extreme desert environment is characterized by prolonged drought, temperature extremes, high radiation and frequent cycles of hydration and dehydration. *S. caninervis* is an ideal organism for the identification and characterization of genes related to abiotic stress tolerance. Reverse transcription quantitative real-time polymerase chain reaction (RT-qPCR) expression analysis is a powerful analytical technique that requires the use of stable reference genes. Using available *S. caninervis* transcriptome data, we selected 15 candidate reference genes and analyzed their relative expression stabilities in *S. caninervis* gametophores exposed to a range of abiotic stresses or a hydration-desiccation-rehydration cycle. The programs geNorm, NormFinder, and RefFinder were used to assess and rank the expression stability of the 15 candidate genes. The stability ranking results of reference genes under each specific experimental condition showed high consistency using different algorithms. For abiotic stress treatments, the combination of two genes (α*-TUB2* and *CDPK*) were sufficient for accurate normalization. For the hydration-desiccation-rehydration process, the combination of two genes (α*-TUB1* and *CDPK*) were sufficient for accurate normalization. *18S* was among the least stable genes in all of the experimental sets and was unsuitable as reference gene in *S. caninervis.* This is the first systematic investigation and comparison of reference gene selection for RT-qPCR work in *S. caninervis*. This research will facilitate gene expression studies in *S. caninervis*, related moss species from the *Syntrichia* complex and other mosses.

## INTRODUCTION

*Syntrichia caninervis* is a desert moss and the dominant bryophyte of the biological soil crusts found in the Gurbantunggut desert of Northwestern China (Zhang, 2005). The Gurbantunggut has a mean annual precipitation of ∼79.5 mm and a mean annual evaporation of 2,606.6 mm (Zhang, 2005; Zhang et al., 2011a). *S. caninervis* has gained particular attention due to its extreme desiccation tolerance (DT; Wood, 2007; Yang et al., 2012) and is closely related to *Tortula ruralis*. *T. ruralis* is a model DT moss and many desiccation-related genes have been isolated and analyzed in this species *T. ruralis* (Chen et al., 2002; Chen and Wood, 2003; Peng et al., 2005). EST data from desiccated and rehydrated *T. ruralis* gametophytes indicated many novel genes exist in this DT species (Wood et al., 1999; Oliver et al., 2004). *S. caninervis,* as compared to *T. ruralis,* is reported to be more tolerant to desiccation stress and have a quicker recovery rate from complete water loss (Oliver et al., 1993; Zhang et al., 2011b). Furthermore, *S. caninervis* is tolerant to multiple stresses including drought, high/low temperature, high radiation and frequent cycles of hydration and dehydration (Yang et al., 2012). *S. caninervis* has been systematically studied at the morphological (Stark et al., 2004, 2005, 2009; Stark and McLetchie, 2006; Zhang et al., 2007; Xu et al., 2009a; Zheng et al., 2011; Tao and Zhang, 2012), physiological (Xu et al., 2009b; Li et al., 2010; Zhang et al., 2011b; Wu et al., 2012) and molecular levels (Yang et al., 2012), and is being developed as another model moss for studying the mechanisms of DT and the identification of stress-related genes.

Reverse transcription quantitative real-time polymerase chain reaction (RT-qPCR) is one of the most widely used technologies for gene expression studies because of its quantitative accuracy, high sensitivity and high-throughput capabilities (Bustin, 2000; Huggett et al., 2005). The utilization of stable reference genes are a prerequisite for RT-qPCR, although the quality and quantity of the mRNA template and reaction efficiency variations also influence the reliability and accuracy of the technique (Vandesompele et al., 2002; Udvardi et al., 2008; Bustin et al., 2009). RT-qPCR data must be normalized with more than one internal reference gene (Hu et al., 2009) and some reports have suggested that at least three reference genes be combined to normalize the results of RT-qPCR (Hong et al., 2010; Jacob et al., 2013). Reference genes may show different stability patterns even within the same plant, and the results of RT-qPCR cannot be extrapolated to other experimental conditions (Exposito-Rodriguez et al., 2008). Consequently, it is recommended that suitable reference genes should be established for each species and tested for specific experimental conditions to ensure their stability (Li et al., 2012).

Studies on reference gene identification and selection for RT-qPCR have focused on humans and other animal model organisms (Shen et al., 2010; Ponton et al., 2011). In plant science, growing efforts have been made in recent years. Stable reference genes have been identified in variety of plants, such as *Arabidopsis* (Lilly et al., 2011), grasses (Hong et al., 2008; Lee et al., 2010), fruits (Clancy et al., 2013; Die and Rowland, 2013; Imai et al., 2014), vegetables (Wan et al., 2010; Xu et al., 2012), commercial agricultural crops (Figueiredo et al., 2013; Wang et al., 2013b), and some desert plants (Li et al., 2012; Shi et al., 2012; Zhu et al., 2013). The development of next generation sequencing technology provides new opportunities to explore the genetic resources from an expanding selection of plants (Czechowski et al., 2005; Hong et al., 2010; Narsai et al., 2010; Demidenko et al., 2011; Feng et al., 2013). Furthermore, with the increasing awareness of importance of suitable reference genes, different statistical algorithms like geNorm (Vandesompele et al., 2002), NormFinder (Andersen et al., 2004), BestKeeper (Pfaﬄ et al., 2004), and RefFinder (Xie et al., 2012) have been developed to determine which reference gene(s) is most suitable for transcript normalization in a given experiment condition for a specific species.

Reference gene identification and selection has been conducted in a variety of plant species, however, studies are limited in non-vascular plants to the moss *Physcomitrella patens* (Le Bail et al., 2013) and the brown algae *Ectocarpus siliculosus* (Kianianmomeni and Hallmann, 2013). To date, no study has been performed to evaluation stable reference gene for a desiccation tolerant moss species in response to abiotic stress. The 18S rRNA (*18S*) has been utilized as a RT-qPCR reference gene in *S. caninervis* (Yang et al., 2012); however a systematic investigation and stability comparison of reference genes has not been done in *S. caninervis*.

In this study, 15 reference gene candidates were selected including 12 traditional reference genes (*ACT*, *ARP*, α*-TUB1*, α*-TUB2*, β*-TUB*, *HIS3*, *18S*, *SPT*, *UBR1*, *UBR2*, *GAPDH1*, *GAPDH2*) and three new reference genes (*CDPK*, *F-BOX*, *SAND*) which demonstrated stable expression in the plants *P. patens* and *Arabidopsis*. The expression stabilities of these 15 genes were evaluated in *S. caninervis* gametophytes subjected to 11 abiotic stress conditions (include one non-stress control) and four samples involved in hydration-desiccation-rehydration process (H-D-R). RT-qPCR data were analyzed using two most widely used algorithms geNorm and NormFinder to determine sets of reference genes suitable for gene expression studies in different experiment conditions. Additionally, a comprehensive reference gene stability analysis tool RefFinder was used to confirm the ranking results obtained from geNorm and NormFinder. This work will facilitate future work on gene expression studies in *S. caninervis* and also benefit other species of the mosses genus such as *T. ruralis.*

## MATERIALS AND METHODS

### PLANT MATERIALS AND TREATMENT

*Syntrichia caninervis* gametophytes were collected from the Gurbantunggut Desert of Xinjiang Uyghur Autonomous Region of China (Fukang County, 44°32′30^′′^N, 88°6′42^′′^E). This sand dune was identified as a permanent research site since 2003 (Wu et al., 2012). The collected moss gametophytes were air-dried and kept in a black bag at room temperature for 1 week.

*Syntrichia caninervis* gametophytes were exposed to 11 different treatments (i.e., stress condition) and a hydration-desiccation-rehydration process to evaluate the stabilities of the tested reference genes. For the hydration-desiccation-rehydration process, dry gametophores were fully hydrated by placing upon MINIQ-filtered water saturated filter paper (8 mL) in glass petri dishes for 24 h at 25°C (150 μm m^-2^ s^-1^; Zhang et al., 2011b), then transfer to clean dishes and dried at room temperature (air dry) for 6 h (∼25°C, RH = 25%; Yang et al., 2012), then rehydrated by transferring to new petri plates and the filter paper was saturated with 8 mL filtered water at 25°C for 2 and 6 h. For abiotic stress treatments, the fully hydrated gametophytes (hydrated for 24 h) were transferred to new petri plates and the filter paper was saturated with 8 mL of one of the following solutions at 25°C: MINIQ-filtered water (control), 20% (w/v) PEG6000 (osmotic stress), 250 mM NaCl (salt stress), 50 mM H_2_O_2_ (oxidative stress), 500 μM CuSO_4_ (metal stress), and 100 μM ABA (exogenous ABA application). For UV exposure, fully hydrated gametophores were exposed to 0.5 w/m^2^ UV-B irradiation. For wounding, fully hydrated gametophores were cut into small pieces with a razor blade. For cold and heat stresses, gametophores were placed in petri plates on water saturated filter paper and incubated at either 4 or 42°C. For the combination of osmotic stress and elevated temperature stress, fully hydrated gametophores were transferred to petri plates saturated with 8 mL of 10% PEG6000 and incubated at 42°C; all the samples were harvested at 6 h after treatment, and gametophyte shoots (removal of rhizoid, i.e., 100 mg FW) were collected for each sample. Harvested samples were flash frozen in liquid nitrogen and stored at –80°C prior to RNA extraction.

### RNA EXTRACTION AND cDNA SYNTHESIS

Total RNA was extracted using TRIzol reagent (Qiagen, USA). Genomic DNA contamination was eliminated using RNase-free DNaseI (Takara, Japan). RNA concentration, purity, and integrity were determined using a NanoDrop ND-2000 spectrophotometer (Thermo Fisher Scientific, USA) and visually assessed via gel electrophoresis (1.2% agarose). Only RNA samples with a 260/280 ratio between 1.8 and 2.1 and 260/230 ratio higher than 1.8 were used for subsequent analyses. First strand cDNA was synthesized from 1 μg total RNA, l μl oligo-dT, 1 μl random hexamers, and 4 μl 5 × Primerscript Buffer using PrimeScript^TM^ RT reagent kit (perfect Real time; Takara, Japan). The reverse transcription was carried out at 37°C for 30 min on a C1000^TM^ Thermal cycler (Bio-Rad, USA) in a final volume of 20 μl, and inactivation of the enzyme was achieved at 85°C for 5 min. All cDNA were stored at –20°C until use.

### REFERENCE GENES SELECTION, PCR PRIMER DESIGN, AND TESTING

Based on previous RT-qPCR reports in the model plants *Arabidopsis* and *P. patens*, we selected 15 reference genes spanning a range of biological functions as reference gene candidates in *S. caninervis*. The genes are: 18S ribosomal RNA (*18S*), actin (*ACT*), actin-related protein (*ARP*), alpha tubulin (α*-TUB1* and α*-TUB2*), beta tubulin (β*-TUB)*, calmodulin-like domain protein kinase (*CDPK*), F-box/kelch-repeat protein (*F-BOX*), glyceraldehyde-3-phosphate dehydrogenase (*GAPDH1* and *GAPDH2*), histone H3 (*HIS3*), SAND protein family (*SAND*), suppressor of Ty (*SPT*), and ubiquitin protein ligase (*UBR1* and *UBR2).* Most of them are well characterized, plant classic reference genes candidates such as *18S*, *ACT,* and α*-TUB* genes. Three are newly identified candidates which have demonstrated good stability in plants, including *SAND*, *CDPK,* and *F-BOX*. The *18S* gene (KJ398837) was cloned previously by our lab, and the other 14 genes were obtained from our transcriptional data of *S. caninervis* (Gao et al., 2014). RT-qPCR primers were designed with Primer Premier 5.0 using the following criteria: amplicon length from 100 to 300 bp and a T_m_ of 58 ± 3°C. The designed primer sets were BLASTed against the local transcriptional data of *S. caninervis* to verify primer specificity. All RT-qPCR experiments conformed to the MIQE guidelines (Bustin et al., 2009). Amplification efficiency (E) was evaluated using a standard curve generated by RT-qPCR using a 10-fold dilution series (1, 1/10, 1/100, 1/1000, 1/10000, 1/100000) over at least four dilution points that were measured in triplicate. Primer specificity was assessed using melting-curve analysis after RT-qPCR and gel electrophoresis analysis of the amplicons. Primer pairs performed well which showed single product and no product amplified in no-template control (NTC) were further sequenced to exclude the amplification of high identity homologs.

### REAL-TIME QUANTITATIVE PCR

cDNA were diluted five times for RT-qPCR. Real-time PCR reactions were carried out in 96-well plates with CFX96 Real-Time PCR Detection System (Bio-Rad, USA) using SYBR *Premix Ex Taq^*TM*^* (Takara, Japan). The reaction mixture consisted of 2 μl 1:5 diluted cDNA samples, 0.4 μl each of the forward and reverse primers (10 μM), 10 μl real-time master mix and 7.2 μl PCR-grade water in a final volume of 20 μl. Two biological replicates for all of the samples and three technical replicates of each biological replicate with a NTC were also used. The RT-qPCR protocol was as follows: 30 s initial denaturation at 95°C, 40 cycles of 94°C for 5 s and 58–61°C for 30 s. To verify the specificity of each primer, a melting-curve analysis was included (65–95°C with fluorescence measured every 0.5°C).

### ANALYSIS OF GENE STABILITY

To rank the stability of the tested genes, the two publicly available tools geNorm (Version 3.5) and NormFinder were used. Additionally, a comprehensive web-based tool RefFinder (http:www.leonxie.com/referencegene.php) was used to integrate and confirm the results obtained using geNorm and NormFinder. These three programs can evaluate the expression stability of reference genes from different aspects. GeNorm is a popular algorithm to determine the most stable reference genes and the optimal number of genes needed for accurate normalization. The raw Cq values were imported into Microsoft Excel and transformed into relative quantities using the formula Q = 2^(mincq^ ^-samplecq)^, then imported into geNorm to analysis gene expression stability. The sample with the highest expression level (the minimum Cq value) was used as a calibrator and was set to 1, subsequently the expression level of other samples were converted to a relative expression. According to the geNorm manual, the expression stability value (M) and pairwise variation value (V) for each reference gene with all other genes were automatically analyzed and ranked according to their expression stability. Then, the optimal number of reference genes for normalization was determined. As suggested by geNorm, the cutoff of M value was set as 1.5, a lower value of average expression stability (M) indicated more stable gene expression, The pairwise variation (Vn/Vn+1) was analyzed to determine the optimal number of reference genes for accurate normalization. The cutoff value was proposed to be 0.15, below which the inclusion of an additional reference genes is not necessary, while it is suggested that this cutoff should not be too strict (Vandesompele et al., 2002). The NormFinder program identified the genes with optimal normalization among a set of candidate genes according to intra and inter variations. The lowest stability value (the least intra and inter-group variations) indicates the most stable expression within the gene set examined. RefFinder is a comprehensive tool which integrates commonly used reference gene evaluation programs together, including geNorm, NormFinder, Bestkeeper, and the comparative delta Ct methods. RefFinder generated the final overall ranking of tested reference genes based on the geometric mean of the weights of every gene calculating by each program. Raw Cq values (untransformed data) were used directly for data importing of RefFinder program.

## RESULTS

### SELECTION OF CANDIDATE REFERENCE GENES AND GENE SEQUENCE ANALYSIS

The cDNA fragments of the 15 reference genes ranged from 480 bp for *SAND* to 2390 bp for *CDPK.* BLASTP demonstrated that all *S. caninervis* references genes had maximum identity with similar deduced polypeptides from *P. patens*, (identity ranged from 58 to 100%), (Table S1). For example, *ScCDPK* share 90% sequence identity with *PpCDPK* (XP_001776407), 62% with *AtCDPK* (NP_196107; Table S1), and 99% identity with *TrCDPK* (GenBank No AAB70706) in desiccation model moss *T. ruralis*. BLASTN demonstrated that *Sc18S* had 97% identity with *Arabidopsis 18S* gene, and shared 90% identity with *18S* genes in both *P. patens* and *T. ruralis*. All cDNA sequences were deposited to the GenBank database under accession numbers KJ398821 to KJ398834.

### VERIFICATION OF PRIMER SPECIFICITY AND EFFICIENCY

The RT-qPCR primer sequences and amplicon characteristics of 15 candidate reference genes are described in **Table 1**. The sequence length was ranged from 117 to 277 bp. The primer efficiency (E) for each primer pair was greater than 90% and varied from 91.6% for *ACT* to 103.2% for *GAPDH1*, and correlation coefficients (R) ranged from 0.992 for *SAND* to 1.0 for *ACT*, *HIS3*, *18S,* and *CDPK*, respectively (**Table 1**). All primer sets were BLAST searched against the non-redundant (nr) database in NCBI (primer-BLAST program) as well as the local *S. caninervis* transcriptome data to verify primer specificity. Primer specificity was further assessed by melting-curves and gel electrophoresis analysis. RT-PCR and RT-qPCR products for each primer pair amplified only a single product of the predicted size, and a single peak was obtained by melting-curve analysis (Figure S1). The amplicons were further sequenced to exclude the amplification of homologs with high identities. These analyses demonstrated that each primer pairs used in this study is specific to the candidate genes.

**Table 1:** Primer sequences and amplicon characteristics of 15 reference genes for RT-qPCR analysis.

Gene symbol	Accession number	Primer sequence (5′→3′)	Length (bp)	Tm (°C)	E (%)	*R*^2^
*ACT*	KJ398821	TCGTGTTGTCTGGAGGATCGTACTCGCTCTTCGCAATCCA	196	86.5	91.6	1.0
*ARP*	KJ398822	GCAAGTAGCGAAGGGTAAATGCCGTATGGTGGAGATG	277	86	94.4	0.994
α*-TUB1*	KJ398823	CGTCGTCTATGATGGCGAAGTTCTTGATCGTTGCCACTGCC	117	86	95.7	0.996
α*-TUB2*	KJ398824	CGGTCATTACACCGTGGGAACCTCTCCAGCAACAGCGAA	154	86	101.0	0.996
β*-TUB*	KJ398825	CTTTGACCTCCCGTGGCTCGTTCGGGATCCACTCAACGA	227	89	98.8	0.998
*HIS3*	KJ398826	AGGAGTGAAGAAGCCCCATCCGAACAGACCCACCAGGTAC	215	87.5	99	1.0
*18S*	KJ398837	GGAGAGGGAGCCTGAGACACCAGACTTGCCCTCCAA	181	84	99.1	1.0
*SPT*	KJ398827	ACTTGGCACATCATCGTCCATTCCGCTGGTTTCATCC	185	84	101.1	0.999
*UBR1*	KJ398828	GCAGGGAGGCATACCTTTCTTATGAGCCCTCTGTTGTTTGGA	117	84.5	101.3	0.995
*UBR2*	KJ398829	AGCTTGTTACTGGCTTTGGGGGAGTTCATCCTGCGTTGC	202	84	100.4	0.999
*GAPDH1*	KJ398830	GCGTTGTTGCTGCCCAGTCAGGCGAGTCCTTCCTTCCAT	146	87	103.2	0.992
*GAPDH2*	KJ398831	GGGCTTCTCAAGGCTGATGTTCCACCACGTAATCAGCACC	134	84.5	98.7	0.995
*CDPK*	KJ398832	AACACTTCAGGTGCCACATAGGCATCATTCAACGAGGACAG	253	85	98.1	1.0
*F-BOX*	KJ398833	CGCCTTCAAAGTCATCATCGGGCAAATCGCCTCACAGTAG	191	88.5	101.9	0.999
*SAND*	KJ398834	AAAGCCTTGGACATGGGAGACGTCGCTTGTGGCATAGAA	263	85.5	100.3	0.992

*Amplicons length (Length), melting temperature (Tm, based on melt curve analysis),amplification efficiency (E), correlation coefficient (R^*2*^)*.

### EXPRESSION PROFILING OF THE CANDIDATE REFERENCE GENES (RT-qPCR ASSAY)

**FIGURE 1:**
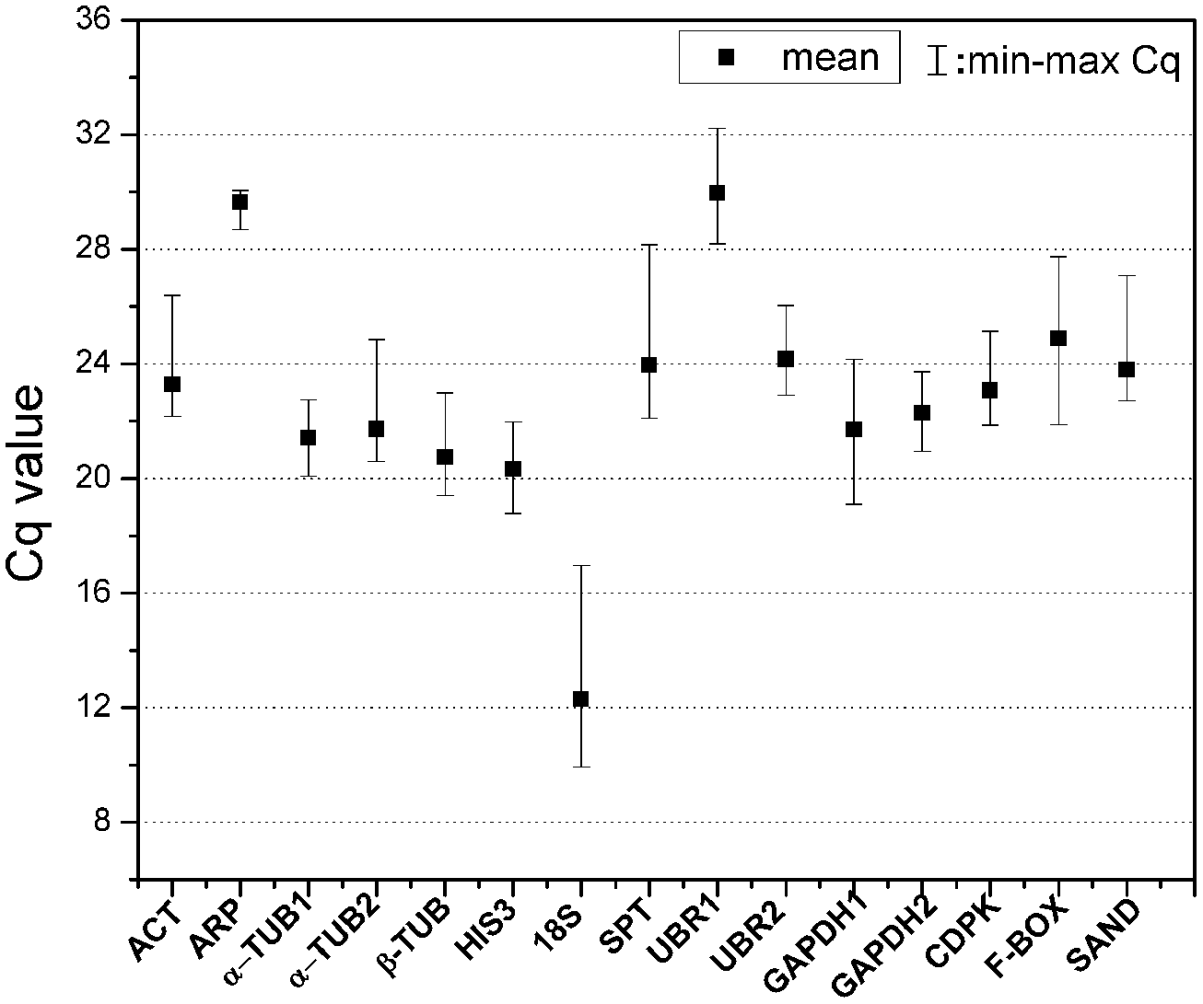
**Expression levels of 15 candidate reference genes in 15 different samples.** The median value and the minimum and maximum Cq of the 15 samples were calculated. Two biological replicates for all of the samples and three technical replicates of each biological replicate were used.

The expression levels of 15 candidate reference genes were determined as quantification cycles (Cq value; **Figure 1**; Table S2). The mean Cq values for reference genes ranged between 12 and 30, with most lying between 20 and 25 across all tested samples. The average Cq value of all the tested samples was 22.8 cycles. *UBR1* had the highest median Cq value (29.98), which indicated relatively low expression. In contrast, *18S* gene was highly expressed compared to the protein coding genes (Cq = 12.3). *ARP*, α*-TUB1*, and *GAPDH2* each had low gene expression variation (below three cycles), while *18S* and *SPT* showed higher gene expression variation (above six cycles; Table S2). The variable transcript abundance of the 15 reference genes demonstrated that different reference genes showed varied expression levels under the same experiment set. Even for the same reference gene, gene expression was varied across different *S. caninervis* samples. This confirmed that no individual gene shows constant expression under all experimental conditions, and stable reference gene selection is necessary for each specific experimental condition in *S. caninervis*.

### EXPRESSION STABILITY ANALYSIS OF THE CANDIDATE REFERENCE GENES

The expression levels of tested reference genes were analyzed and ranked using three programs, geNorm, NormFinder, and RefFinder individually.

#### GeNorm analysis

**FIGURE 2:**
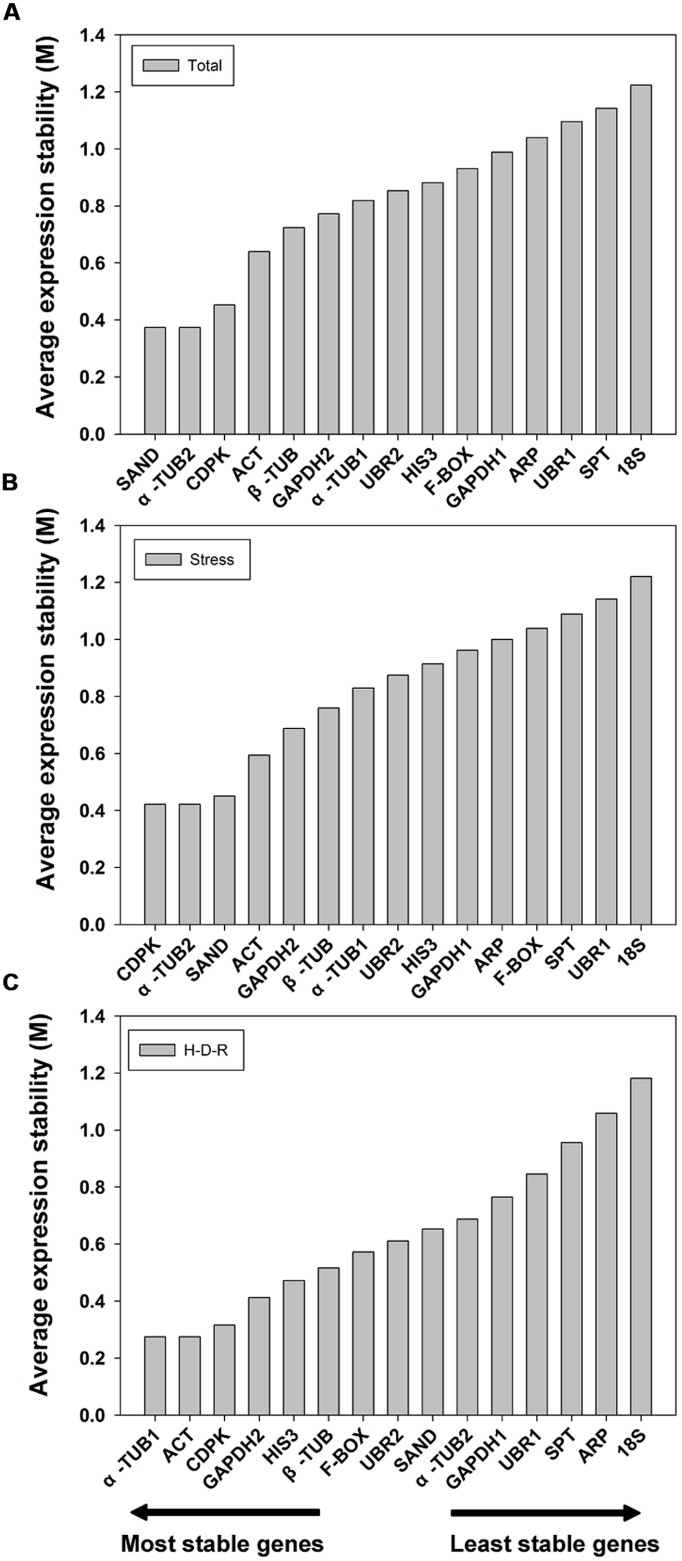
**Expression stability values (M) and ranking of the candidate reference genes based on geNorm algorithm.** The cutoff M value was proposed to be 1.5, a lower M value indicated more stable gene expression. The most stable genes are listed on the left and the least stable on the right. **(A)** All tested samples, **(B)** abiotic stresses, **(C)** hydration-desiccation-rehydration process.

GeNorm ranked the candidate genes based on the assumption that the expression ratio of two ideal reference genes should be constant throughout different test samples (Vandesompele et al., 2002). The expression stability value (M) and pairwise variation value (V) for each reference gene with all other genes were automatically analyzed and ranked. It is recommend using an M value below the threshold of 1.5 (Vandesompele et al., 2002; the lower the M value, the higher the gene’s expression stability). In our analysis, all 15 candidate reference genes had an M value less than 1.5 (**Figure 2**) which indicated that all these genes were acceptable stable reference gene candidates. For all the tested samples, α*-TUB2* and *SAND* were the most stable two genes, while *18S* was the least stable. The gene stability ranking (from the most stable to the least stable) across all the samples was: α*-TUB2*/*SAND* > *CDPK* > *ACT* > β*-TUB* > *GAPDH2* > α*-TUB1* > *UBR2* > *HIS3* > *F-BOX* > *GAPDH1* > *ARP* > *UBR1* > *SPT* > *18S* (**Figure 2A**). Similar results were obtained for abiotic stress treated samples, with slight changes in the order of ranking. The gene stability ranking (from the most stable to the least stable) for stress samples were: α*-TUB2*/*CDPK* > *SAND* > *ACT* > *GAPDH2* > β*-TUB* > α*-TUB1* > *UBR2* > *HIS3* > *GAPDH1* > *ARP* > *F-BOX* > *SPT* > *UBR1* > *18S* (**Figure 2B**). For the H-D-R process, different ranking results were obtained as compared to the tested samples and stress-treated samples (from the most stable to the least stable): α*-TUB1*/*ACT* > *CDPK* > *GAPDH2* > *HIS3* > β*-TUB* > *F-BOX* > *UBR2* > *SAND* > α*-TUB2* > *GAPDH1* > *UBR1* > *SPT* >*ARP* > *18S* (**Figure 2C**).

#### NormFinder analysis

**FIGURE 3:**
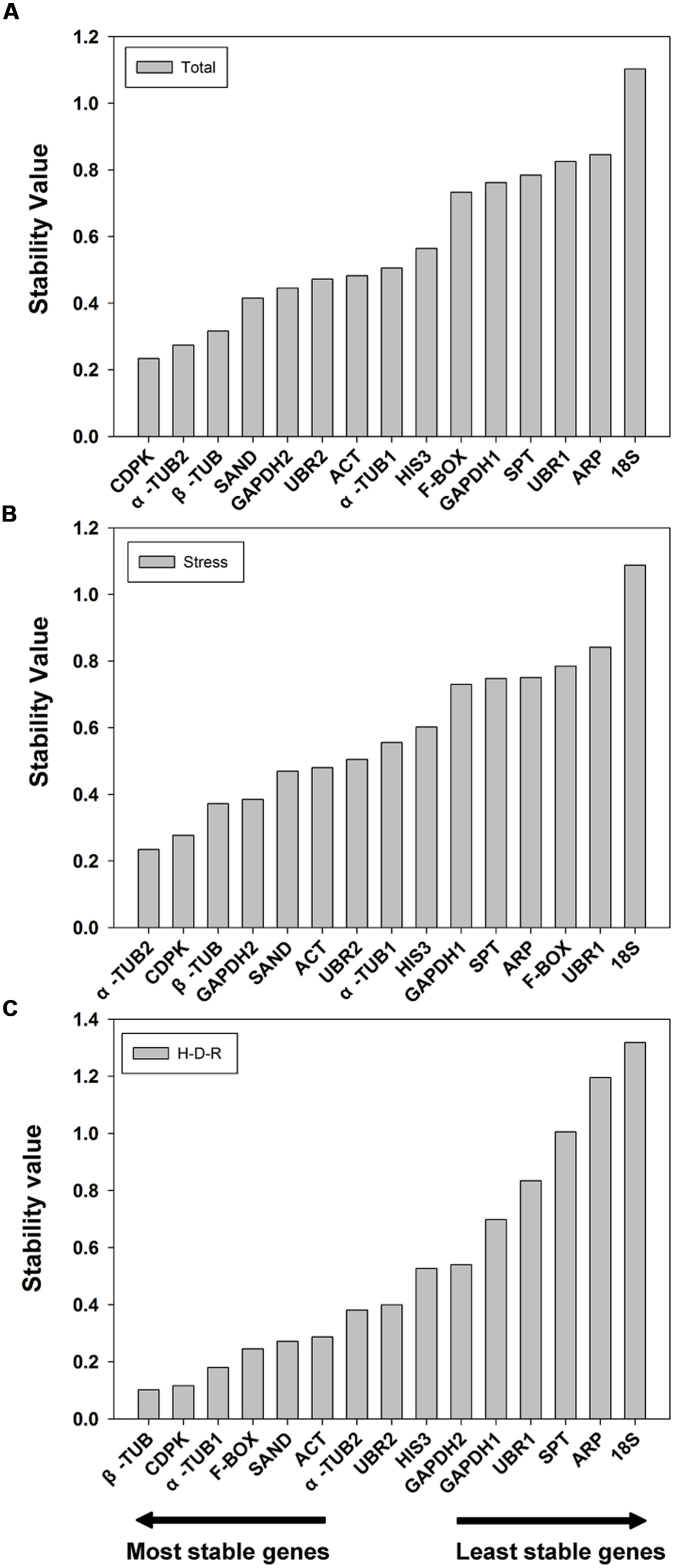
**Stability value and ranking of the candidate reference genes based on NormFinder algorithm.** The lowest stability value indicates the most stable expression within the gene set examined. The most stable genes are listed on the left and the least stable on the right. **(A)** All tested samples, **(B)** abiotic stresses, **(C)** hydration-desiccation-rehydration process.

The 15 candidate reference genes were further evaluated using NormFinder method. NormFinder evaluates the stability of reference genes based on the expression variations of candidate reference genes. The program calculates a stability value for each reference gene and the lower stability value indicated the higher stability (Andersen et al., 2004). NormFinder analysis was performed without grouping. The results are shown in **Figure 3**. For all tested samples, NormFinder demonstrated that *CDPK* was the most stable (ranked third by geNorm) followed by α*-TUB2*, β*-TUB*, *SAND*. Similar to geNorm, *18S* was the least stable gene. The stability ranking order (from the most stable to the least stable) was: *CDPK* > α*-TUB2* > β*-TUB* > *SAND* > *GAPDH2* > *UBR2* > *ACT* > α*-TUB1* > *HIS3* > *F-BOX* > *GAPDH1* > *SPT* > *UBR1* > *ARP* > *18S* (**Figure 3A**). For abiotic stress, NormFinder suggested that α*-TUB2* and *CDPK* were the most stable genes, and *18S* and *UBR1* were the least stable. These results are consistent with those obtained by geNorm. The stability ranking order (from the most stable to the least stable) was: α*-TUB2* > *CDPK* > β*-TUB* > *GAPDH2* > *SAND* > *ACT* > *UBR2* > α*-TUB1* > *HIS3* > *GAPDH1* > *SPT* > *ARP* > *F-BOX* > *UBR1* > *18S* (**Figure 3B**). For H-D-R process, β*-TUB* was the most stable gene. Notably, both by geNorm and NormFinder demonstrated that *18S*, *ARP*, *SPT*, *UBR1,* and *GAPDH1* were the five least stable genes in response to hydration-desiccation-rehydration. The stability ranking order (from the most stable to the least stable) was: β*-TUB* > *CDPK* > α*-TUB1* > *F-BOX* > *SAND* > *ACT* > α-*TUB2* > *UBR2* > *HIS3* > *GAPDH2*> *GAPDH1*> *UBR1*>*SPT*>*ARP* > *18S* (**Figure 3C**).

#### RefFinder analysis

**FIGURE 4:**
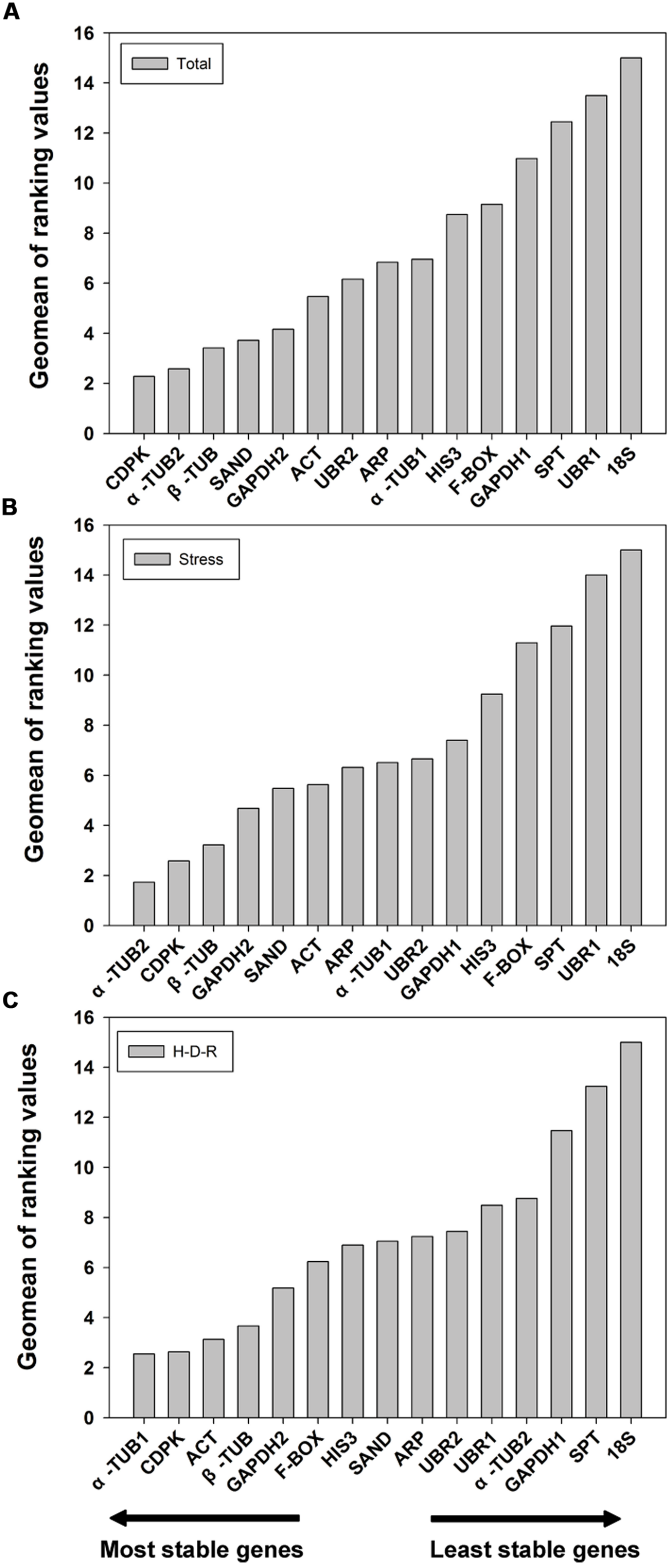
**Geomean of ranking values of the candidate reference genes based on RefFinder algorithm.** RefFinder is a comprehensive tool which generated the final overall ranking of tested reference genes based on the geometric mean of the weights of every gene calculating by each program including geNorm, NormFinder, Bestkeeper, and the comparative delta Ct methods. The most stable genes are listed on the left and the least stable on the right. **(A)** All tested samples, **(B)** abiotic stresses, **(C)** hydration-desiccation-rehydration process.

The results obtained from geNorm and NormFinder were further confirmed using the comprehensive ranking platform RefFinder (Xie et al., 2012). RefFinder is a web-based tool which integrates four current computing programs to compare and re-rank the tested reference genes based on the geometric mean of the weights of every single gene calculating by each program. The final ranking results are shown in **Figure 4**. For all the tested samples, a similar ranking order was obtained using RefFinder as compared to NormFinder (from the most stable to the least stable): *CDPK* > α*-TUB2* > β*-TUB* > *SAND* > *GAPDH2* > *ACT* > *UBR2* > *ARP* > α*-TUB1* > *HIS3* > *F-BOX* > *GAPDH1* > *SPT* > *UBR1* > *18S* (**Figure 4A**). For stress samples, the ranking result obtained from RefFinder was also more consistent with NormFinder. The ranking order (from the most stable to the least stable) was: α*-TUB2* > *CDPK* > β*-TUB* > *GAPDH2* > *SAND* > *ACT* > *ARP* > α*-TUB1* > *UBR2* > *GAPDH1* > *HIS3* > *F-BOX* > *SPT*> *UBR1*> *18S* (**Figure 4B**). For the H-D-R process, the ranking order suggested by RefFinder was more similar to geNorm. The top three stable genes were α*-TUB1*, *ACT,* and *CDPK* (**Figure 4C**). The ranking order was: α*-TUB1* > *CDPK* > *ACT* > β*-TUB* > *GAPDH2* > *F-BOX* > *HIS3* > *SAND* > *ARP* > *UBR2* > *UBR1* >α*-TUB2 > GAPDH1* > *SPT* > *18S*.

The stability of reference genes were assessed and ranked using three different algorithms (i.e., geNorm, NormFinder, and RefFinder; **Table 2**). The rankings are consistent between the algorithms, especially for the top six most stable genes (Italics) and the four least stable genes (underlined). For example, geNorm, NormFinder, and RefFinder all demonstrated that α*-TUB2*, *CDPK*, β*-TUB* were the most stable genes, while *18S*, *UBR1*, *SPT* were always low ranked and were the least stable genes for various abiotic stress treatment samples.

**Table 2:** Consensus stability ranking of the reference genes evaluated by geNorm, NormFinder, and RefFinder tools.

Experimental sample sets	The six most stable genes	Most stable combination	The four least stable genes
Total	α*-TUB2 CDPK*β*-TUB SAND GAPDH2 ACT*	α**-TUB2+ CDPK**	18S UBR1 SPT ARP
Stress	α*-TUB2 CDPK*β*-TUB GAPDH2 SAND ACT*	α**-TUB2+ CDPK**	18S UBR1 SPT F -BOX
H-D-R	α*-TUB1 CDPK*β*-TUB ACT F -BOX GAPDH2*	α**-TUB1 **+** CDPK**	18S ARP SPT UBR1

*Most stable two reference genes were bold and the four least stable genes were underlined*.

### OPTIMAL NUMBER OF REFERENCE GENES FOR RT-qPCR NORMALIZATION

**FIGURE 5:**
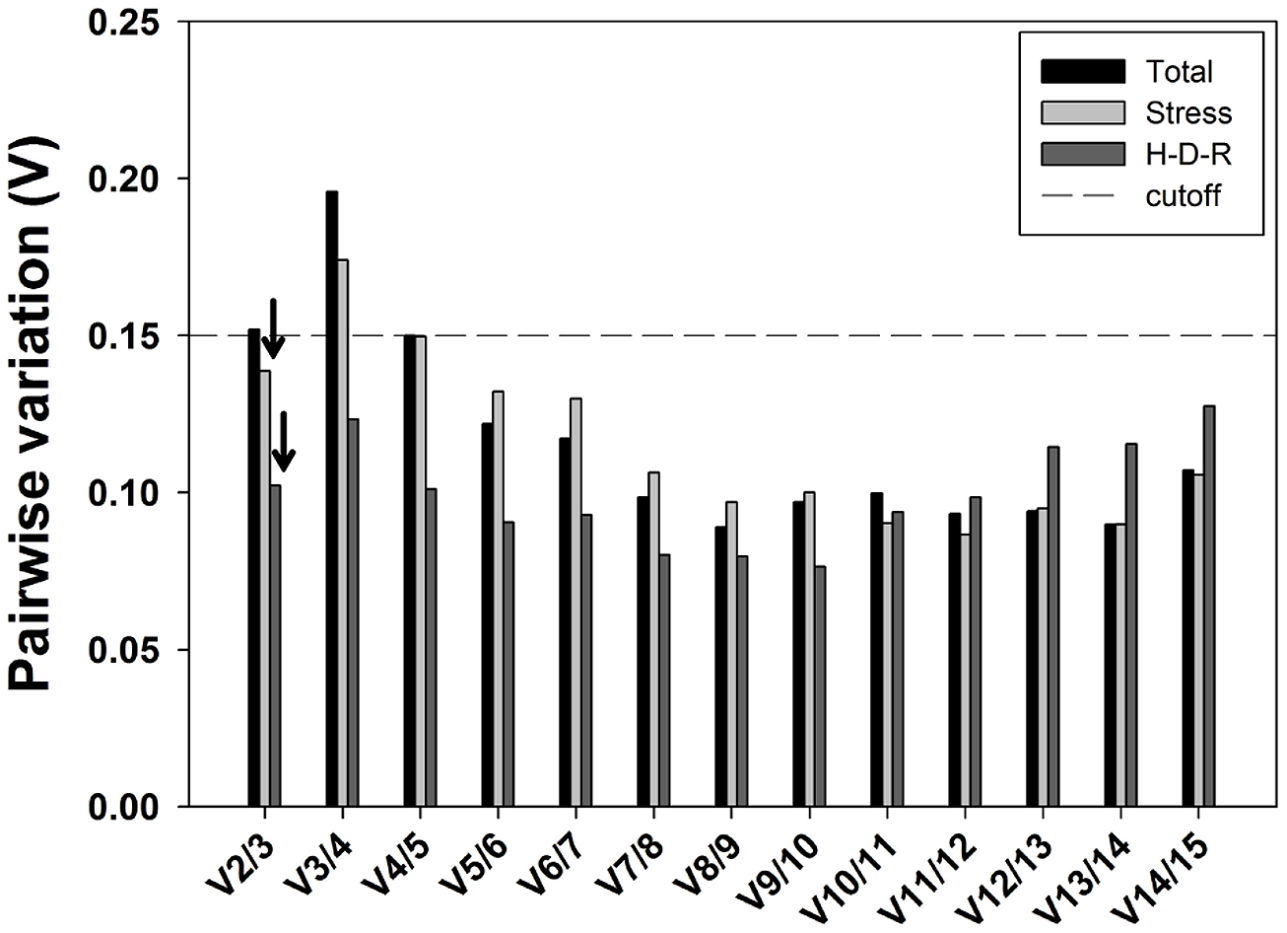
**Pairwise variation (Vn/Vn**+**1) analysis of the candidate reference genes.** The pairwise variation (Vn/Vn+1) was analyzed based on geNorm algorithm to determine the optimal number of reference genes for accurate normalization. The cutoff value was proposed to be 0.15, below which the inclusion of an additional reference genes is not necessary. Arrows indicated the optimal number of reference genes.

The optimal number of reference genes for accurate normalization of RT-qPCR was also recommended by geNorm through calculating the pairwise variation Vn/Vn+1 value using 0.15 as the proposed cutoff value (Vandesompele et al., 2002). A Vn/Vn+1 value less than 0.15 means the inclusion of the n+1 reference genes is not required, and the top n reference genes are adequate for accurate normalization of RT-qPCR results. When considering all samples, pairwise variation analysis demonstrated that V2/3 exceeded the recommend cutoff value (0.15; **Figure 5**) and the inclusion of the third a nd fourth stable reference genes (i.e., *SAND* and *ACT*) were needed to improve the accuracy of normalization. For stress and hydration-desiccation-rehydration process conditions, the V2/3 value was 0.139 and 0.102, respectively, which indicated that the two most stable reference genes were sufficient for reliable normalization. For the H-D-R process subset, all pairwise variation values (from V2/3 to V14/15) were below the proposed cutoff 0.15. For stress conditions, the combination of *CDPK* and α*-TUB2* are sufficient for normalization while the combination of α*-TUB1* and *CDPK* are sufficient for the H-D-R process.

## DISCUSSION

### REFERENCE GENE IDENTIFICATION AND SELECTION IN MOSSES

Reverse transcription quantitative real-time polymerase chain reaction is a powerful technology for gene expression studies, and the utilization of suitable reference genes is a prerequisite to ensure reliable and accurate data. Numerous studies have documented the selection of reference genes in various plants including *Arabidopsis* (Czechowski et al., 2005; Remans et al., 2008; Hong et al., 2010; Lilly et al., 2011) and crops such as soybean (Libault et al., 2008; Hu et al., 2009; Kulcheski et al., 2010). In this regard, the model moss *P. patens* has been extensively studied and the vast majority of RT-qPCR gene expression studies employ either *ACT* (Yamawaki et al., 2011; Cui et al., 2012; Liu et al., 2013) or *UBQ* (Harries et al., 2005; Dittrich and Devarenne, 2012) as a single reference gene. Recently, reference gene selection in *P. patens* has focused on hormone treatment and the comparison of developmental stages (Le Bail et al., 2013). Gene expression studies in the moss *T. ruralis* have employed *18S* as an internal control (Zeng and Wood, 2000; Chen et al., 2002; Zeng et al., 2002). Our initial RT-qPCR experiments in *S. caninervis* employed *18S* (Yang et al., 2012) and no other reference genes were tested or compared. However, a growing number of studies have demonstrated that *18S*, *ACT,* and *UBQ* genes were not stable in many species under non-standard experimental condition (Jian et al., 2008; Lovdal and Lillo, 2009; Yang et al., 2010; Lilly et al., 2011). In *P. patens*, Wang et al. (2012) reported that *ACT* and *GAPDH* genes are up-regulated by abiotic stress. Overall, reference gene research in mosses lags behind other major plant groups. To date, few studies have undertaken a systematic comparison and selection of reference genes in mosses or within moss gametophores exposed to abiotic stress. Very few moss genes deposited in the database is the main reason for the limitation of reference gene study in mosses, more work including reference gene isolation and selection need to increase efforts for mosses gene study especially for extreme stress tolerant species.

### REFERENCE GENES STABILITY IN *S. caninervis*

Recent studies demonstrated that the expression of some classic reference genes, such *ACT* (Lovdal and Lillo, 2009; Lilly et al., 2011), *18S* (Die et al., 2010; Yang et al., 2010), and *GAPDH* (Li et al., 2012; Wang et al., 2012) can vary greatly and are unsuitable for gene normalization (particularly in response to abiotic stress). In this study, *ACT* and *GAPDH2* genes showed good stability ranking (within the top six stable genes), while *18S* was always the least stable gene under three tested experimental conditions. Some studies support the idea that new reference genes can be more stably expressed under specific conditions as compared with classic ones (Czechowski et al., 2005; Libault et al., 2008). In the present study, three new reference genes *CDPK*, *F-BOX,* and *SAND* were tested. Similar to soybean (Libault et al., 2008), our results showed that the *CDPK* gene was one of the top two stable genes under all experimental conditions as determined by each of the algorithms. In contrast, the *F-BOX* gene which is reported to be a stable reference gene (Czechowski et al., 2005; Libault et al., 2008) was always amongst the least stable genes in our analysis. The *SAND* gene is also reported to be a stable reference gene (Czechowski et al., 2005; Demidenko et al., 2011; Lilly et al., 2011; Zhu et al., 2013) and in our study was suitable for analyzing abiotic stress samples.

### COMPREHENSIVE ASSESSMENT OF REFERENCE GENE STABILITY USING MULTIPLE EVALUATION TOOLS

Increasing awareness of the importance of suitable reference genes has lead to the development of different statistical algorithms to determine which reference gene(s) is best suited for transcript normalization under a given experiment condition for a specific species. GeNorm, NormFinder, Bestkeeper are three widely applied algorithms used for the assessment of reference gene stability. Since different algorithms may obtain differing stability rankings, one challenge is to identify the most stable (and therefore most suitable) reference gene. RefFinder, was developed to provide a final comprehensive ranking of reference genes based on the geometric mean of the weights of every gene calculated by each program (Xie et al., 2012). Jacob et al. (2013) recommend more than two algorithms should be used for reference gene stability evaluation. Bestkeeper cannot analyze more than 10 reference genes and was therefore excluded from this study (Pfaﬄ et al., 2004). The results from geNorm, NormFinder, and RefFinder were consistent (i.e., ranking of the six most stable and four least stable reference genes were identical), although the specific rankings of each reference gene varied. This indicated that the results obtained from these three software application were sufficient for accurate validation in this study.

### TRANSCRIPTOME-BASED SYSTEMATIC SELECTION IS AN IMPORTANT STRATEGY FOR REFERENCE GENE STUDY

The number of reference genes evaluated in plants (especially for non-model plants) is limited and usually does not exceed eight sequences (Yang et al., 2010; Xu et al., 2012; Wang et al., 2013a) due in part to the limited availability of species-specific gene sequences. With access to large-scale datasets, the evaluation and selection of reference genes have proliferated in model plants such as *Arabidopsis* (Czechowski et al., 2005) and rice (Narsai et al., 2010). As an important model moss for DT research and valuable anti-stress genes discovery, the first large-scale transcriptome data for *S. caninervis*, consisting of 92,240 unigenes (Gao et al., 2014), has been recently characterized and has served as the source of the reported gene selection. In this study, we perform a systematic comparison and selection of reference genes suitable for a wide range of experimental conditions from the desert plant *S. caninervis*. Our results demonstrate that transcriptome sequencing data is a useful source for candidate reference genes screening and represents an important strategy for large-scale reference gene selection for non-model plants.

We performed the first systematic selection of reference genes in desert moss *S. caninervis*. Our results reinforce the idea that reference gene stability should test in specific plant species, under particular experimental conditions, in similar tissues and evaluated with multiple programs. The stably expressed reference genes identified in this study will facilitate future work on gene expression studies in mosses and *Syntrichia* ssp. in particular.

## Conflict of Interest Statement

The authors declare that the research was conducted in the absence of any commercial or financial relationships that could be construed as a potential conflict of interest.
