# Supplementary material for: Characterization of reference genes for RT-qPCR in the desert moss Syntrichia caninervis in response to abiotic stress and desiccation/rehydration
Source: Front Plant Sci. 2015 Feb 5;6:38. doi: 10.3389/fpls.2015.00038 (PMC4318276; doi:10.3389/fpls.2015.00038)
Supplement: Supplementary file 1 [file Table_1.DOCX]

**Supporting information**

**Table S1.** **Gene description and amino acids sequence identity with model moss *P.patens* and model plant Arabidopsis of 14 reference gene candidates from transcriptome data.**

| Gene symbol | Gene  description | Function | Unigene number | *P.patens* homolog and Identity (aa%) | *A.thaliana* homolog and identity (aa%) |
| --- | --- | --- | --- | --- | --- |
| *ACT* | actin | Cytoskeletal structure protein | Unigene2690 | AAQ88110.1  368/376(98%) | NP_566988.1  352/377(93%) |
| *ARP* | actin-related protein 9 | Actin filament assemble | Unigene12272 | XP_001756914.1  294/333(88%) | NP_974876.1  153/326(47%) |
| *a-TUB1* | alpha tubulin | Cytoskeletal structure protein | Unigene157 | XP_001777110.1  192/200(96%) | BAH19954.1  181/186(97%) |
| *a-TUB2* | alpha tubulin | Cytoskeletal structure protein | Unigene40296 | XP_001777081.1  258/261(99%) | NP_171974.1  245/261(94%) |
| *β-TUB* | beta-tubulin | Cytoskeletal structure protein | Unigene1729 | XP_001766554.1  440/443(99%) | NP_568437.1  404/430(94%) |
| *HIS3* | histone H3 | Chromatin structure | Unigene40303 | XP_001752178.1  136/136(100%) | NP_001078516.1  137/141(97%) |
| *SPT* | transcription elongation factor SPT6 | Transcription elongation | Unigene22597 | XP_001756686.1  179/308(58%) | NP_001077775.1  126/308(41%) |
| *EF1 a* | Translation elongation factor | Translation | Unigene6739 | XP_001763194.1 416/429(97%) | NP_200847.1  401/429(93%) |
| *UBR1* | ubiquitin protein ligase | Protein degradation | Unigene18464 | XP_001772932.1  102/177(58%) | NP_565676.4  92/179(51%) |
| *UBR2* | E3 ubiquitin protein ligase RIN2 | Protein degradation | Unigene48966 | XP_001765176.1  379/439(86%) | NP_194253.2  216/446(48%) |
| *GAPDH1* | glyceraldehyde-3-phosphate dehydrogenase | Glycolysis | Unigene38934 | XP_001771209.1  349/378(92%) | BAH57068.1  296/349(85%) |
| *GAPDH2* | glyceraldehyde-3-phosphate dehydrogenase | Glycolysis | Unigene39027 | CAC80387.1  293/315(93%) | NP_178071.1  243/315(77%) |
| *CDPK* | calmodulin-like domain  protein kinase | Signal transduction | Unigene68078 | XP_001776407.1  518/576(90%) | NP_196107.1  377/609(62%) |
| *F-BOX* | F-box/kelch-repeat protein | Signal transduction biological  processes | Unigene6323 | XP_001781265.1 179/251(71%) | NP_197075.1  73/259(28%) |
| *SAND* | Sand family protein | Signal transduction | Unigene87449 | XP_001785228.1  32/37(86%) | NP_029426.1  55/99(56%) |

**Table S2. Cq values of 15 *S. caninervis* candidate reference genes among 15 tested samples.**

| Gene | Mean | Min | Max | Mean-min | Max-mean | Max-min |
| --- | --- | --- | --- | --- | --- | --- |
| *ACT* | 23.29 | 22.17 | 26.39 | 1.11 | 3.11 | 4.22 |
| *ARP* | 29.64 | 28.69 | 30.06 | 0.95 | 0.42 | 1.37 |
| *α-TUB1* | 21.41 | 20.07 | 22.75 | 1.34 | 1.34 | 2.68 |
| *α-TUB2* | 21.73 | 20.60 | 24.86 | 1.14 | 3.12 | 4.26 |
| *β-TUB* | 20.74 | 19.40 | 23.00 | 1.34 | 2.26 | 3.60 |
| *HIS3* | 20.34 | 18.78 | 21.98 | 1.57 | 1.63 | 3.20 |
| *18S* | 12.30 | 9.94 | 16.96 | 2.36 | 4.66 | 7.02 |
| *SPT* | 23.96 | 22.11 | 28.17 | 1.85 | 4.21 | 6.06 |
| *UBR1* | 29.98 | 28.21 | 32.23 | 1.77 | 2.25 | 4.03 |
| *UBR2* | 24.17 | 22.91 | 26.04 | 1.26 | 1.87 | 3.13 |
| *GAPDH1* | 21.72 | 19.10 | 24.16 | 2.62 | 2.44 | 5.06 |
| *GAPDH2* | 22.29 | 20.93 | 23.73 | 1.35 | 1.45 | 2.80 |
| *CDPK* | 23.07 | 21.86 | 25.14 | 1.21 | 2.07 | 3.28 |
| *F-BOX* | 24.89 | 21.87 | 27.74 | 3.02 | 2.85 | 5.87 |
| *SAND* | 23.82 | 22.71 | 27.08 | 1.11 | 3.26 | 4.37 |

**Figure S1. Verifying the specificity of RT-qPCR amplification.** (**A**) Agarose gel (2.0%) showing the amplified products of the reference genes from qRT-PCR at the expected sizes. (**B**) Dissociation curves of 15 reference genes showing single peaks for each including three technical replicates for each of the 15 cDNA pools of test samples.
