# Supplementary figures and images for: Characterization of reference genes for RT-qPCR in the desert moss Syntrichia caninervis in response to abiotic stress and desiccation/rehydration
Source: Front Plant Sci. 2015 Feb 5;6:38. doi: 10.3389/fpls.2015.00038 (PMC4318276; doi:10.3389/fpls.2015.00038)

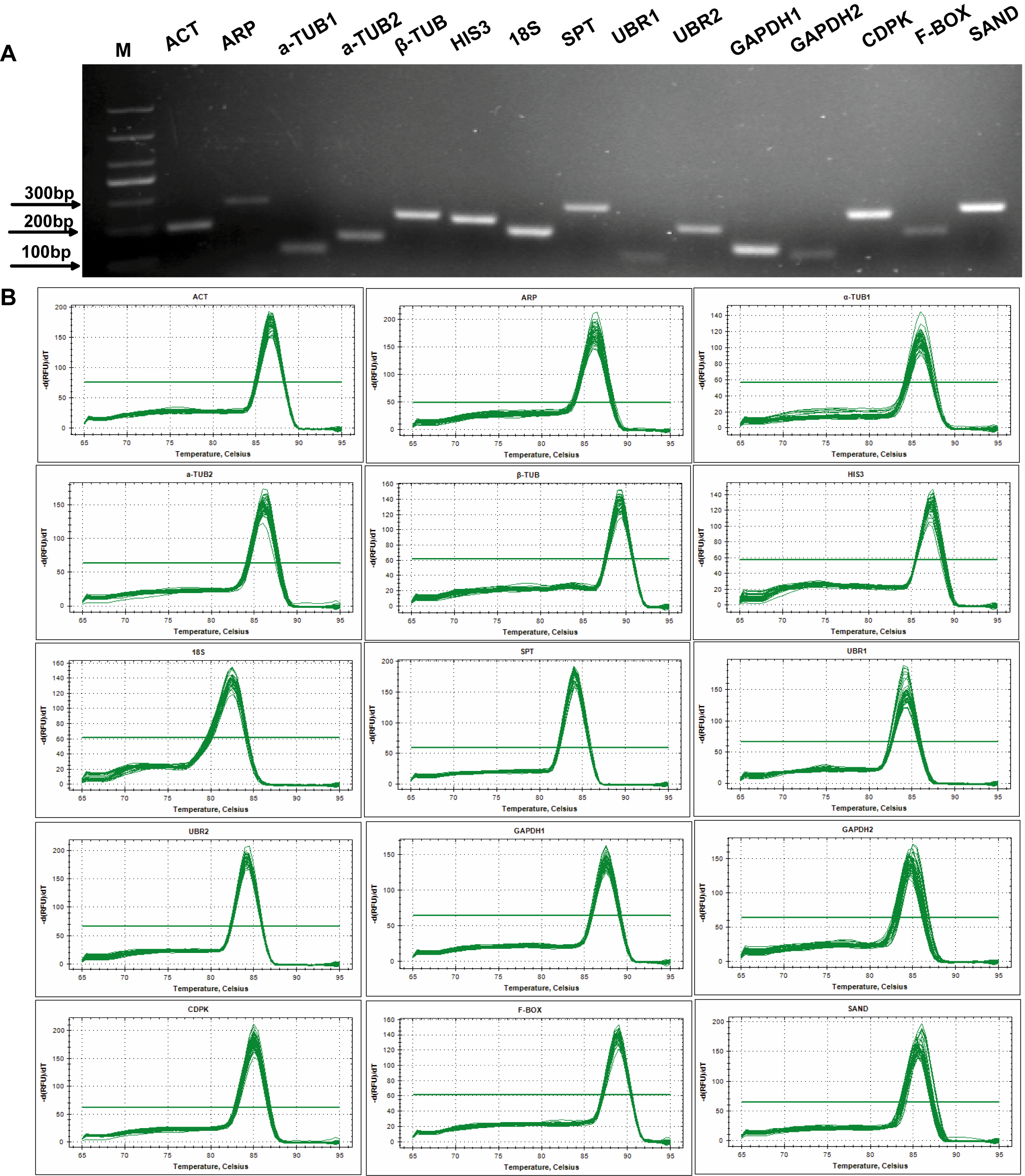

Supplement: Supplementary file 2 [file Image_1.TIF]
